# Crosstalk between hydrogen sulfide and nitric oxide in endothelial cells

**DOI:** 10.1111/jcmm.12077

**Published:** 2013-06-07

**Authors:** Zaid Altaany, Guangdong Yang, Rui Wang

**Affiliations:** a Department of Biology, Lakehead UniversityThunder Bay, Ontario, Canada; b The School of Kinesiology, Lakehead UniversityThunder Bay, Ontario, Canada

**Keywords:** Hydrogen sulfide, Nitric oxide, Endothelial cells, eNOS, CSE, Cystathionine gamma-lyase

## Abstract

Hydrogen sulfide (H_2_S) and nitric oxide (NO) are major gasotransmitters produced in endothelial cells (ECs), contributing to the regulation of vascular contractility and structural integrity. Their interaction at different levels would have a profound impact on angiogenesis. Here, we showed that H_2_S and NO stimulated the formation of new microvessels. Incubation of human umbilical vein endothelial cells (HUVECs-926) with NaHS (a H_2_S donor) stimulated the phosphorylation of endothelial NO synthase (eNOS) and enhanced NO production. H_2_S had little effect on eNOS protein expression in ECs. L-cysteine, a precursor of H_2_S, stimulated NO production whereas blockage of the activity of H_2_S-generating enzyme, cystathionine gamma-lyase (CSE), inhibited this action. CSE knockdown inhibited, but CSE overexpression increased, NO production as well as EC proliferation. LY294002 (Akt/PI3-K inhibitor) or SB203580 (p38 MAPK inhibitor) abolished the effects of H_2_S on eNOS phosphorylation, NO production, cell proliferation and tube formation. Blockade of NO production by eNOS-specific siRNA or nitro-L-arginine methyl ester (L-NAME) reversed, but eNOS overexpression potentiated, the proliferative effect of H_2_S on ECs. Our results suggest that H_2_S stimulates the phosphorylation of eNOS through a p38 MAPK and Akt-dependent pathway, thus increasing NO production in ECs and vascular tissues and contributing to H_2_S-induced angiogenesis.

## Introduction

Hydrogen sulfide and NO are known gasotransmitters that contribute to many physiological functions 1. These gaseous messengers can be produced endogenously to respond to diverse physiological and pathophysiological stimuli 1. In ECs, H_2_S can be generated from L-cysteine by the enzymatic action of CSE (EC 4.4.1.22) 2. Hydrogen sulfide-induced relaxation of vascular tissue was partially reduced by the removal of the vascular endothelium or in the presence of L-NAME (an inhibitor of NO synthase) 3. Nitric oxide can be generated in ECs from L-arginine by eNOS (EC 1.14.13.39) 4. Being a homodimeric protein, the activation of eNOS is dependent on intracellular calcium (Ca^2+^) level and other cofactors like nicotinamide adenine dinucleotide phosphate (NADPH), tetrahydrobiopterin (BH_4_), flavin adenine dinucleotide (FAD) and flavin mononucleotide (FMN) 5. The activity of eNOS is affected by many post-translational modification mechanisms, such as phosphorylation on multiple amino acids like Ser-1179/1177 (bovine/human) and Thr-495 residues 6, 7, whereas eNOS protein can be self-inhibited by high concentrations of NO through *S*-nitrosylation 8. Due to its reducing capability, H_2_S may reduce NO to form a thiol-sensitive molecule *S*-nitrothiols (RSNO) 9. Conversely, H_2_S has been found to reduce RSNO to release NO from GSNO (S-Nitrosoglutathione) 10. Moreover, H_2_S and NO interact on each other's catalysing enzymes; NO donor increases the expression and activity of CSE in cultured aortic smooth muscle cells (SMCs) 3. In rat vascular SMCs, H_2_S had no direct effect on NO production, but it augmented interleukin-induced NO production, and this effect was related to increased iNOS expression (inducible NOS) 11. NaHS (a H_2_S donor) treatment reduced eNOS activity and expression but not of nNOS (neuronal NOS) and iNOS in isolated rat aortas and HUVECs 12. NaHS inhibited eNOS-catalysed conversion of [^3^H]-arginine to [^3^H]-citrulline 13. NaHS also inhibited iNOS expression and NO production in macrophage cells (RAW264.7) 14. Na_2_S selectively augmented NO production in chronically ischaemic tissues, by influencing iNOS and nNOS expression and stimulating nitrite reduction to NO *via* xanthine oxidase (XO) under hypoxic condition 15.

The angiogenic crosstalk between H_2_S and NO in ECs has been unclear. Our present study showed that the pro-angiogenic effect of H_2_S appears to be regulated by both a NO-dependent and an independent mechanism, whereas NO effect on angiogenesis is partially dependent on H_2_S. We demonstrated that H_2_S stimulated NO release by increasing eNOS phosphorylation *via* a p38 MAPK and Akt-dependent mechanism, which contributes to the stimulatory effect of H_2_S on EC proliferation and angiogenesis.

## Materials and methods

### Cell culture and chemicals

Human umbilical vein endothelial cells-derived EA.hy 926 cells were kindly provided by Dr. Cora-Jean S. Edgell 16 (University of North Carolina, USA). The cells were cultured in Dulbecco's modified eagles medium (DMEM) without ferric nitrate (Sigma-Aldrich, Oakville, ON, Canada), containing penicillin (100 U/ml), streptomycin (100 μg/ml) and 10% (v/v) foetal bovine serum. The primary aortic ECs were isolated from the aorta of 10- to 12-week-old C57BL/6J/129 mice, as previously described 17. Aortic ECs were cultured in a medium containing 20% FBS, 100 U/ml penicillin-G, 100 μg/ml streptomycin, 2 mM L-glutamine, 25 mM HEPES (pH 7-7.6), 100 μg/ml heparin, 100 μg/ml endothelial cell growth supplement (ECGS) and DMEM (Sigma-Aldrich). The nature of ECs was confirmed using endothelial-specific markers CD31 (Santa Cruz Biotechnology, Santa Cruz, CA, USA) and eNOS (Cell Signaling Technologies, Beverly, MA, USA) by Western blot, and endothelial tube formation using Matrigel assay (BD Biosciences, Mississauga, ON, Canada; data not shown). The culture medium was changed every 2 days and ECs between passages 3 and 5 were used.

### Measurement of NO production

Total nitrate/nitrite concentrations were measured by conversion of nitrate to nitrite after incubating supernatants with nitrate reductase (10 U/ml) and NADPH (5 mM) for 1 hr at 37°C. The total nitrite was measured with a Griess assay kit (Promega, Madison, WI, USA) using a reference sodium nitrate standard curve 18. The results obtained with the Griess assay have also been validated by the diaminofluorescein fluorophore system (DAF-FM), which can be deacetylated by intracellular esterases and further reacts with NO to form a fluorescent benzotriazole (DAF fluorescence; Invitrogen, Burlington, ON, Canada). Endothelial cells were incubated with 5 μM DAF-FM for 30 min. at 37°C. The cells were washed to remove excess dye, replaced with fresh medium and observed under a fluorescent microscope as previously described 19. To detect the production of NO in aortic tissues, isolated aortas were incubated with DAF-FM (5 μM) at 37°C in Kreb's buffer and then rapidly removed and frozen at −20°C. Aortic tissue samples were embedded in optimal cutting temperature (OCT) compound until frozen, and sectioned using Leica CM1850 UV microtome-cryostat (Leica Biosystems, Concord, ON, Canada). The tissue blocks were cut into 10-μm thick sections and observed under a fluorescent microscope 20.

### Gene knockdown and overexpression

Endothelial cells were seeded in 6-well plates and cultured until they reached 70–80% confluence. The cells were then transfected with specific siRNA to knockdown CSE or eNOS gene (50 nM). Negative siRNA was used as transfection control (50 nM), using Lipofectamine™ RNAi-MAX transfection reagent according to the manufacturer's instruction (Invitrogen). Overexpression experiments were carried out with plasmid DNA containing CSE cDNA (pIRES2-EGFP, 4.0 μg) or eNOS cDNA (pcDNA 3.1 eNOS-GFP, 4.0 μg). Mock empty vector was used as transfection control (Addgene, Cambridge, MA, USA) 21–23 using Lipofectamine™ 2000. Forty-eight hours after transfection, the cells or media were collected and evaluated by Western blot or Griess assay analysis.

### Western blot analysis

Cultured cells were collected and incubated in a lysis buffer containing 0.5 M EDTA, 1 M Tris-Cl (pH 7.4), 0.3 M sucrose and a protease inhibitors mixture (Sigma-Aldrich). The cell extracts were sonicated three times (5–10 sec./each) on ice using a cell sonicator (Sonic Dismemrator Model 100, Fisher Scientific, Ottawa, ON, Canada) 23. Cellular extracts were separated by centrifugation at 14,000 × *g* for 15 min. at 4°C. Supernatants were collected, and the same amounts of proteins were separated on 10% SDS-polyacrylamide gels and blotted onto nitrocellulose membranes (Pall Corporation, Pensacola, FL, USA). All primary antibody incubations were performed at 4°C overnight. The antibody dilution for phospho-eNOS (Ser1177), eNOS, phospho-ERK, ERK, phospho-p38 MAPK, p38 MAPK, phospho-Akt (S473) and Akt was at 1:1000 (Cell Signaling Technologies). Anti-CSE antibody was used at 1:5000 (Proteintech Group, Chicago, IL, USA), and anti-β-actin antibody was at 1:10000 (Sigma-Aldrich). The membranes were stripped using a buffer containing 100 mM β-mercaptoethanol, 2% SDS and 62.5 mM Tris-HCl (pH 6.8) at 50°C for 30 min. Membranes were visualized using enhanced chemiluminescence Western blotting system (GE Healthcare, Piscataway, NJ, USA). Densitometric quantification was performed using Alpha Digi Doctor Software (Richardson, TX, USA). The protein bands were quantified and normalized against either β-actin or total form levels of the target protein, and expressed as a percentage relative to the controls (equals 100%). The phosphorylation level is defined as the ratio between the phosphorylated target proteins and their total forms and expressed in the summarized bar graphs as the percentage of the untreated controls.

### Capillary-like tube formation assay

The Matrigel matrix gel was thawed overnight at 4°C on ice and then added to pre-chilled culture dishes and allowed to polymerize at 37°C for 1 hr. Endothelial cells (2 × 10^4^ cells) were incubated with different agents in 500 μl DMEM and then seeded onto the surface of Matrigel (BD Biosciences). After 12 hrs, the formation of capillary-like structure was imaged by light microscope. The total lengths of tube-like structures per field were measured using image analysis software (NIH Image software- Image J).

### Cell proliferation assay

Cells were counted using automated cell counter TC10™ from BioRad (Mississauga, ON, Canada) and seeded into 96-well plates (1 × 10^4^ cells/well). After 24 hrs of initial seeding, cells were incubated with DMEM serum-free medium for overnight 23. The proliferation rates were evaluated by 5-bromo-2′-deoxyuridine (BrdU) incorporation assay according to the manufacturer's instructions (EMD Biosciences, San Diego, CA, USA).

### Microvessel formation assay

Cystathionine gamma-lyase knockout (KO) mice were generated as described previously 2. Eight-week-old male CSE-KO and wild-type (WT) mice were sacrificed, and aorta were rapidly cleaned off adipose tissues and blood. Aorta were cut into rings (length, ∼3 mm) and implanted in a fibrin gel obtained by adding 400 μl of a fibrinogen solution (3 mg/ml) and thrombin (1.5 U/ml); (Sigma-Aldrich). The fibrin gels were given 30 minutes to solidify before different treatments were applied. As a control, the effect of medium alone was assayed, and quantitative evaluation of new microvessels was carried out after 72 hrs 24. All animal experiments were conducted according to the Care and Use of Laboratory Animals Guide (NIH Publication No. 85-23, revised 1996) and approved by Lakehead University Animal Care Committee, Canada.

### Statistical analysis

All data were expressed as mean ± SEM. Each data point represented at least three to four independent experiments. Statistical comparisons were evaluated using Student's *t-*test. Values of *P* < 0.05 were considered statistically significant.

## Results

### H_2_S-induced NO production in ECs

**Fig. 1:**
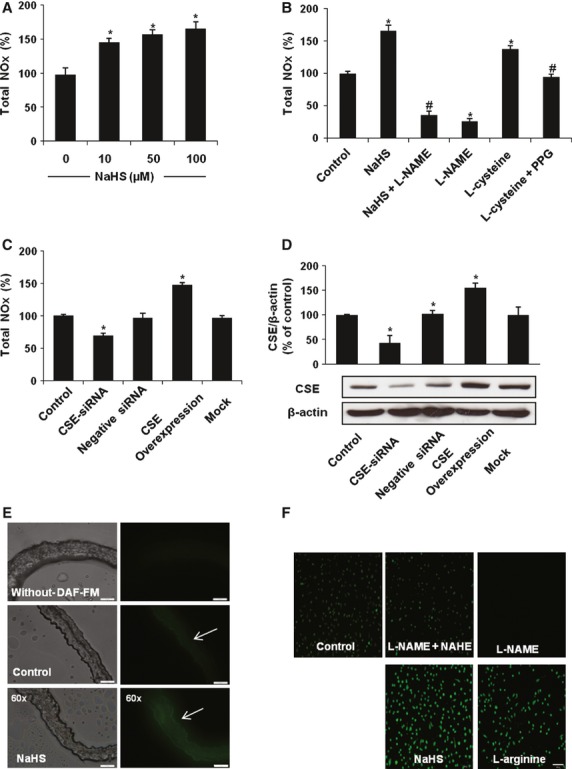
H_2_S stimulated NO production in endothelial cells (ECs) and aortic tissues. (**A**) The effect of NaHS on NO production in ECs detected by Griess assay, *n* = 4, *P < 0.05 *versus* control. (**B**) The effects of NO synthase (NOS) inhibitor L-NAME (200 μM, 1 hr), cystathionine gamma-lyase (CSE) inhibitor PPG (10 mM, 4 hrs), NaHS (100 μM, 30 min) and L-cysteine (6 mM, 30 min.) on NO production detected by the Griess assay, *n* = 3–4, *P < 0.05 *versus* control, #*P* < 0.05 *versus* NaHS or L-cysteine-treated groups. (**C**) The effects of CSE knockdown or overexpression on NO production assessed by the Griess assay. (**D**) The efficiency of CSE knockdown or overexpression, determined by Western blot, *n* = 3–4, *P < 0.05 *versus* control. The effect of NaHS (100 μM) and L-arginine (1 mM) treatment on NO production in isolated aortic tissues (scale bar: 50 μm) (**E**) and cultured ECs **(F)** using diaminofluorescein fluorophore system (DAF-FM) fluorescent probe (scale bar: 200 μm), *n* = 3–4.

Stimulation of ECs with NaHS for 30 min. increased NO production over a concentration range from 10 to 100 μM (Fig. 1A). The effect of NaHS on NO production was blocked when cells were pre-treated with NOS inhibitor Nω-L-NAME (Fig. 1B). NaHS-induced increase in NO production was further confirmed in primarily cultured mouse ECs (Figure S1). Nitro-L-arginine methyl ester treatment also significantly reduced NO production. We next determined the effect of L-cysteine (H_2_S precursor) on NO production. L-cysteine pre-treatment stimulated NO production in ECs (Fig. 1B). However, blocking of CSE activity by PPG reversed L-cysteine effect (Fig. 1B). Cystathionine gamma-lyase knockdown using CSE-specific siRNA significantly reduced CSE protein level and attenuated NO production in comparison with the cells transfected with negative siRNA. Moreover, CSE overexpression significantly elevated CSE expression level and resulted in an increase in NO level (Fig. 1C and D). Nitric oxide data was further confirmed by DAF-FM fluorescence dye showing that NaHS treatment stimulated NO release in aortic tissues and ECs (Fig. 1E and F).

**Fig. 2:**
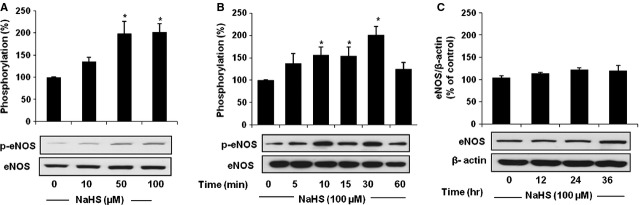
H_2_S stimulated the phosphorylation of endothelial NO synthase (eNOS) in endothelial cells (ECs). (**A**) The effect of NaHS treatment on eNOS phosphorylation. ECs were starved in Dulbecco's modified eagles medium (DMEM) medium free of serum for 24 hrs and treated with different concentrations of NaHS for 30 min. Western blot analysis was conducted using anti-phospho-eNOS and anti-total eNOS antibody, *n* = 3–4, *P < 0.05 *versus* control. (**B**) Time-dependent effect of NaHS treatment on the phosphorylation of eNOS. ECs were treated with NaHS (100 μM) for different periods (0–60 min.). At the end of each time-point, cells were collected and proteins lysates were analysed by Western blot, *n* = 3–4, *P < 0.05 *versus* control. (**C**) The effect of NaHS treatment on eNOS expression level in ECs. The ECs were treated with NaHS (100 μM) for 12–36 hrs, and then cells were collected and proteins were subjected to Western blot analysis. *n* = 3–4, *P < 0.05 *versus* control.

NaHS (50 and 100 μM) treatment markedly increased the phosphorylation of eNOS in ECs (Fig. 2A). The stimulatory effect of NaHS on eNOS phosphorylation was time dependent, and the increase in phosphorylated eNOS appeared at 10 min., peaked at 30 min., and gradually declined to baseline over the period of 1-hr NaHS exposure (Fig. 2B). NaHS treatment up to 36 hrs had no significant effect on eNOS expression level (Fig. 2C).

### The role of p38 MAPK/Akt in H_2_S-induced eNOS phosphorylation and NO production

**Fig. 3:**
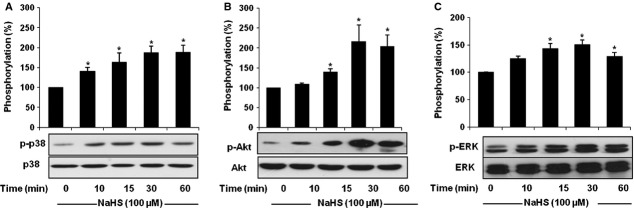
H_2_S-induced phosphorylation of p38 MAPK, Akt and ERK. Endothelial cells (ECs) were treated with NaHS (100 μM) for different times (0–60 min.). At the end of each time-point, cells were collected and proteins lysates were analysed by Western blot, using antibodies specific for the phosphorylated and total forms of (**A**) p38 MAPK, (**B**) Akt, and (**C**) ERK. Data were normalized to total protein level, *n* = 3–4, *P < 0.05 *versus* control.

**Fig. 4:**
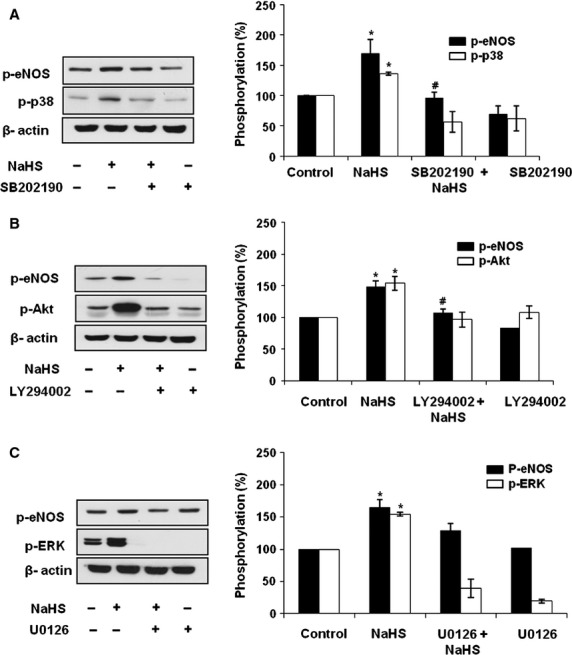
H_2_S-stimulated endothelial NO synthase (eNOS) phosphorylation is dependent on p38 MAPK and Akt. Endothelial cells (ECs) were pre-treated with (**A**) SB203580 (10 μM), (**B**) LY294002 (10 μM), and (**C**) U0126 (10 μM) for 1 hr and then treated with NaHS (100 μM) for 30 min. Cell lysates were harvested and the level of phosphorylated forms of p38 MAPK, Akt, ERK and eNOS were measured by Western blot. *n* = 3, *P < 0.05 *versus* control, #*P* < 0.05 *versus* NaHS-treated group.

**Fig. 5:**
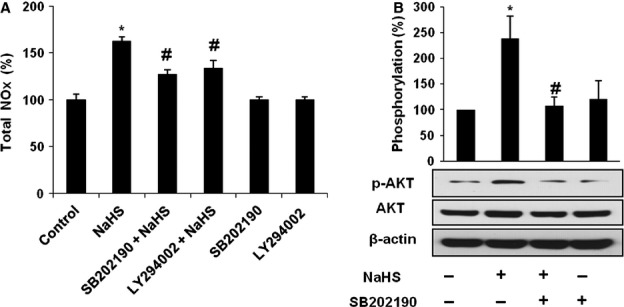
Crosstalk between p38 MAPK and Akt in H_2_S-induced NO production. The p38 MAPK inhibitor inhibited Akt and NO production induced by H_2_S. (**A**) Endothelial cells (ECs) were pre-treated with either SB203580 (10 μM) or LY294002 (10 μM) for 1 hr, and then treated with NaHS (100 μM) for 30 min. At the indicated time-point, the NOx generation was assessed by Griess assay, *n* = 3–4, *P < 0.05 *versus* control, #*P* < 0.05 *versus* NaHS. (**B**) The phosphorylated Akt was measured by Western blot after pre-treatment with SB203580 (10 μM) for 1 hr and NaHS (100 μM) treatment for 30 min., *P < 0.05 *versus* control, *n* = 3–4.

Diverse kinases such as Akt, p38-MAPK kinase and ERK are important for NO production and signalling activation 25–27. To elucidate the signalling pathways involved in H_2_S-induced eNOS phosphorylation and the NO production, we examined the roles of Akt, ERK and p38 MAPK in H_2_S-stimulated NO production. Treatment with NaHS at 100 μM enhanced the phosphorylation of p38 MAPK, Akt and ERK to different levels (Fig. 3). SB202190 (a p38 MAPK inhibitor) and LY294002 (a PI3K/Akt inhibitor), but not U0126 (an inhibitor of ERK), significantly reduced H_2_S-induced phosphorylation of eNOS (Fig. 4). We further found that the stimulatory effect of NaHS on NO production was decreased by the same treatments (SB202190 or LY294002), and neither SB202190 nor LY294002 alone had any detectable effect on NO production (Fig. 5A). In addition, p38 MAPK inhibition by SB202190 attenuated the NaHS-induced phosphorylation of Akt (Fig. 5B), indicating that p38 MAPK might regulate the upstream signalling cascade that leads to Akt activation. These results suggest that p38 MAPK and Akt are required for NO activation by H_2_S.

### The role of NO in H_2_S-induced EC proliferation and angiogenesis

**Fig. 6:**
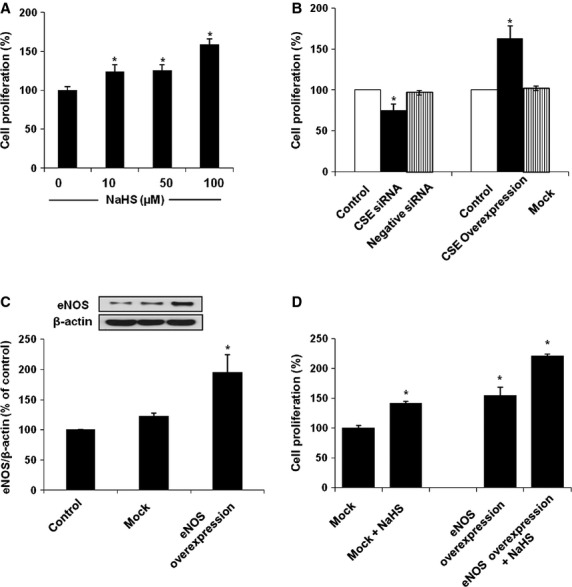
H_2_S-stimulated endothelial cell (EC) proliferation. (**A**) The effects of NaHS treatment on EC proliferation assessed using BrdU proliferation assay. *n* = 3, *P < 0.05 *versus* control. (**B**) The effects of cystathionine gamma-lyase (CSE) knockdown or overexpression on EC proliferation. *n* = 3, *P < 0.05 *versus* control. (**C**) The efficiency of endothelial NO synthase (eNOS) overexpression in ECs detected by Western blot, *n* = 3, *P < 0.05 *versus* Mock. (**D**) The effect of eNOS overexpression on EC proliferation. *n* = 3, *P < 0.05 *versus* Mock.

NaHS significantly induced EC proliferation (Fig. 6A). To show the effect of endogenously produced H_2_S, CSE knockdown with a siRNA approach attenuated cell proliferation. The knockdown of CSE significantly attenuated the proliferation of EC by about 25% compared with the control group (Fig. 6B). We also found that CSE knockdown significantly decreased, but NaHS induced a similar and comparable increase, in the proliferation of primarily cultured mouse ECs (Figure S2). The CSE overexpression stimulated EC proliferation (Fig. 6B). Next, we study the effect of NO on proliferation. The overexpression of eNOS stimulated cell proliferation, which was strengthened by NaHS treatment (Fig. 6C and D).

**Fig. 7:**
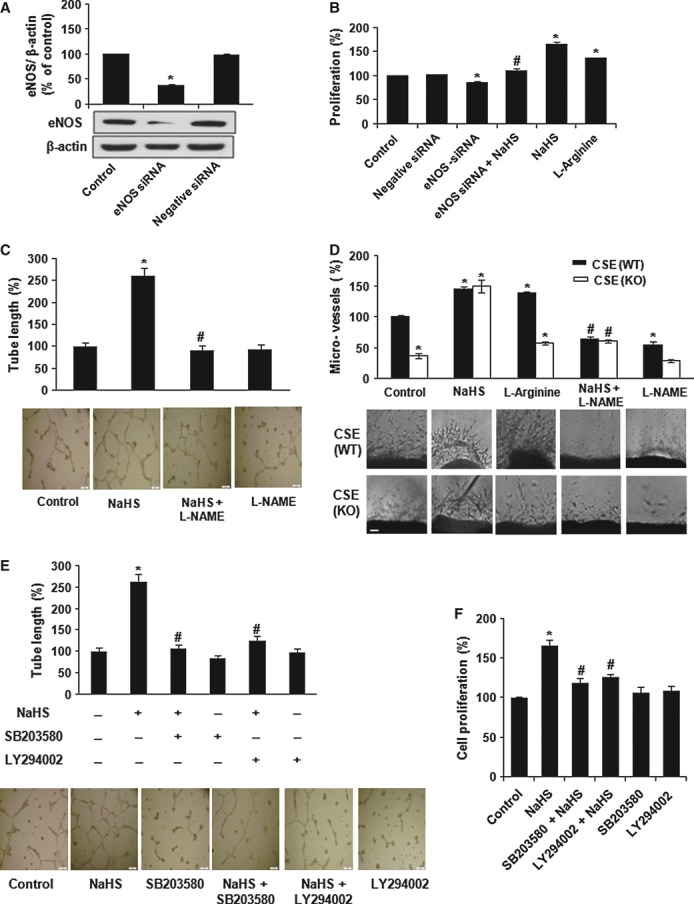
H_2_S interacts with NO to stimulate endothelial cell (EC) proliferation and angiogenesis. (**A**) The efficiency of endothelial NO synthase (eNOS) knockdown transfection in EC detected by Western blot. *n* = 3-4, *P < 0.05 *versus* control. (**B**) The effects of eNOS-knockdown (eNOS siRNA, 50 nM), NaHS (100 μM), and L-arginine (1 mM) treatments on EC proliferation evaluated by BrdU assay. *n* = 3-4, *P < 0.05 *versus* control, #*P* < 0.05 *versus* NaHS-treated group. (**C**) H_2_S-NO interaction on EC tube formation. The effects of NaHS (100 μM) and L-NAME (200 μM) on tube formation of ECs (Scale Bar: 500 μm). (**D**) The effects of L-NAME (200 μM), L-arginine (1 mM) and NaHS (100 μM) on angiogenesis (scale bar: 200 μm), *n* = 3–4 mice for each group, *P < 0.05 *versus* control, #*P* < 0.05 *versus* NaHS-treated group. (**E**) The involvements of p38 MAPK and Akt in EC proliferation and tube formation. ECs were pre-treated with p38 MAPK inhibitor SB202190 (10 μM) and Akt inhibitor LY294002 (10 μM) for 1 hr, and treated with NaHS (100 μM) for 30 min. Cells (2 × 10^4^ cells) were seeded on Martigel for 12 hrs to assist the formation of capillary-like structure (scale bar, 500 μm). (**F**) Cells were pre-treated with LY294002 or SB202190 and NaHS. The cells were cultured for 24 hrs for measurement of proliferation rate using BrdU proliferation assay. *n* = 3–4, *P < 0.05 *versus* control.

We then determined whether H_2_S and NO can interact to regulate angiogenesis. Hydrogen sulfide-induced EC proliferation was attenuated by eNOS knockdown (Fig. 7A and B), whereas treatment with NO precursor L-arginine (1 mM) or NaHS (100 μM) alone significantly increased EC proliferation (Fig. 7B). Furthermore, NaHS (100 μM) treatment significantly increased the capillary-like tube formation of EC compared with the untreated cells (Fig. 7C). NaHS-induced increase in tube formation was significantly attenuated by co-treatment with L-NAME (200 μM), whereas L-NAME treatment alone had no significant effect on tube formation (Fig. 7C). The aortic tissues from CSE-KO mice showed a markedly decreased formation of new microvessels compared with WT mice. After treating the embedded aortic rings with NaHS, the sporting of vascular neogenesis was significantly increased in both CSE-KO and WT mice with markedly higher levels in CSE-KO mice (Fig. 7D). Similar to the effect of NaHS, L-arginine (a NO precursor) stimulated vascular neogenesis in both CSE-KO and WT mice (Fig. 7D). Furthermore, the pro-angiogenic effects of H_2_S on aortic rings from both CSE-KO and WT mice were inhibited by L-NAME treatment (Fig. 7D). Nitro-L-arginine methyl ester treatment inhibited new vessel formation from WT aortic rings, but not that from CSE-KO aortic rings (Fig. 7D). Treatment of EC with NaHS (100 μM) increased the capillary-like tube formation, and co-treatment with a p38 or Akt inhibitor (SB202190 or LY294002) significantly reduced the H_2_S effect (Fig. 7E). However, treatment of EC with LY294002 or SB202190 alone had no significant effect on tube formation (Fig. 7E). LY294002 or SB202190 blocked the proliferation induced by H_2_S, and neither LY294002 nor SB202190 alone had any detectable effect (Fig. 7F), demonstrating that p38 MAPK and Akt are responsible for H_2_S-induced EC proliferation and angiogenesis.

## Discussion

Gasotransmitters play important roles in angiogenesis 28–30. Angiogenesis is important for the development of the cardiovascular system and sustaining blood supplies, wound healing and fetus development 31–35. In our present study, we found that H_2_S can interact with NO to induce angiogenesis of both cloned EC line and freshly isolated primary mouse ECs. The mechanisms for H_2_S action are mainly ascribed to the stimulation of the p38 MAPK/Akt and eNOS phosphorylation, which was followed by increased NO production.

Phosphorylation activates eNOS 30. In our study, the phosphorylation of p38 MAPK precedes the phosphorylation of Akt in the H_2_S signalling cascade, which was confirmed when inhibition of p38 MAPK abolished H_2_S-induced phosphorylation of Akt. We also found that H_2_S activated ERK phosphorylation with a time course similar to that for p38 MAPK activation. However, the inhibition of ERK did not affect H_2_S-stimulated NO production. By altering the phosphorylation of eNOS, H_2_S regulated NO production in ECs. Our observation is consistent with another recent finding by Predmore *et al*. 36 who demonstrated that Na_2_S (150 μM) treatment stimulated NO production in bovine arterial ECs. While these authors illustrated the H_2_S-dependent Akt mechanism that stimulates NO production, the involvement of other kinases, like p38 MAPK, or the synergistic partnership between H_2_S and NO in angiogenesis were not addressed. Conversely, it has been reported that a high concentration of NaHS (300–3000 μM) significantly inhibited the activity of recombinant bovine eNOS 13. It is worthy noted here that NaHS at this high concentration range unlikely bears physiological relevance.

We explored the possible interaction between H_2_S and NO in angiogenesis regulation. *Ex vivo* aortic explants isolated from CSE-KO mice showed a remarkable decrease in vascular neogenesis when compared to WT mice. L-arginine treatment stimulated angiogenesis in the WT mice and to a lesser extent, in the CSE-KO mice. On the other hand, L-NAME treatment reduced new vessel formation in WT mice, and this inhibitory effect was not significant in CSE-KO mice, suggesting that the angiogenic effect of NO might be mediated through H_2_S biosynthesis. Cystathionine gamma-lyase overexpression stimulated EC proliferation, whereas CSE knockdown reversed this effect. Interestingly, we found that the pro-angiogenic effect of H_2_S was partially attenuated in the presence of eNOS inhibitor L-NAME, or after eNOS knockdown using siRNA. Taken together, our results suggest that both gasotransmitters are required for optimal angiogenic activity, yet angiogenesis still proceeds in the presence of either H_2_S or NO alone albeit to a reduced degree. Previous studies had reported that H_2_S and NO can mediate angiogenesis without much knowledge about the H_2_S–NO interaction on angiogenesis 24, 37. Recently, one study reported that a mutually dependent relationship between H_2_S and NO is important for physiological control of different vascular function 38. Our study used different angiogenesis model (CSE-KO mice *versus* rat) and experimental conditions, and we found that H_2_S and NO, alone or combined, can cause angiogenesis. Hydrogen sulfide-stimulated angiogenesis was partially but not completely inhibited by NO blockage, whereas in CSE-KO mice, NO treatment stimulated angiogenesis but to a reduced level. The exact molecular mechanism underlying H_2_S-mediated NO pro-angiogenic response is not clear.

**Fig. 8:**
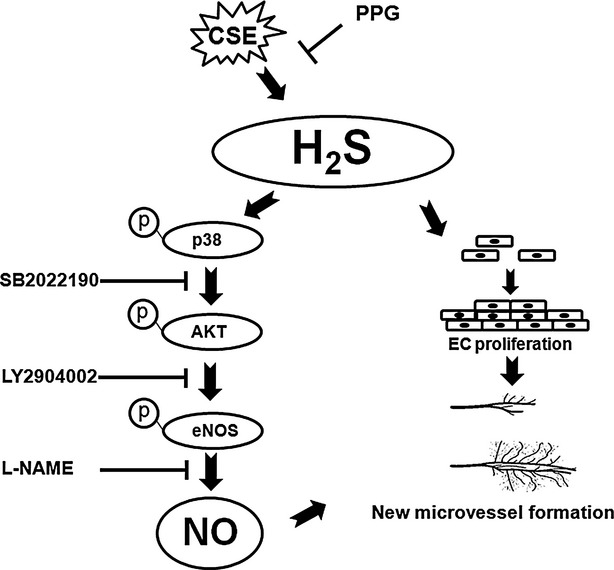
Schematic representation of proposed pathways of H_2_S-induced NO production and angiogenesis.

In summary, our studies demonstrate that H_2_S promotes NO production in ECs *via* the activation of a cascade of phosphorylation events, starting from p38 MAPK, Akt to eNOS. Hydrogen sulfide promotes EC tube formation, proliferation and angiogenesis by NO-dependent and independent mechanisms as outlined in Figure 8. Thus, H_2_S may be a key regulator for angiogenic signalling pathways, whether they required NO or not. The elucidation of the H_2_S–NO relationship in the vascular biology would improve our understanding of the pathogenic mechanisms for cardiovascular disease in general and angiogenic-related diseases in particular.

## Supporting information

**Figure S1** The effect of H_2_S on NO production in primarily cultured mouse aortic endothelial cells. (**A**) The effects of CSE siRNA knockdown and NaHS treatment on NO production detected by Griess assay, *n* = 3, **P* < 0.05 *versus* control, #*P* < 0.05 *versus* NaHS-treated group. (**B**) The effects of NaHS and L-NAME treatment on NO production detected by diaminofluorescein diacetate–based probes (DAF-FM), scale bars: 200 μm.

**Figure S2** The pro-proliferative effects of H_2_S and NO on primarily cultured mouse aortic endothelial cells. (**A**) The effects of CSE siRNA, eNOS siRNA and NaHS treatments on EC proliferation assessed using BrdU proliferation assay, *n* = 3, **P* < 0.05 *versus* control, #*P* < 0.05 *versus* NaHS-treated group (**B**) The efficiency of CSE siRNA and eNOS siRNA in ECs detected by Western blot.
