# Supplementary figures and images for: Crosstalk between hydrogen sulfide and nitric oxide in endothelial cells
Source: J Cell Mol Med. 2013 Jun 7;17(7):879–88. doi: 10.1111/jcmm.12077 (PMC3822893; doi:10.1111/jcmm.12077)

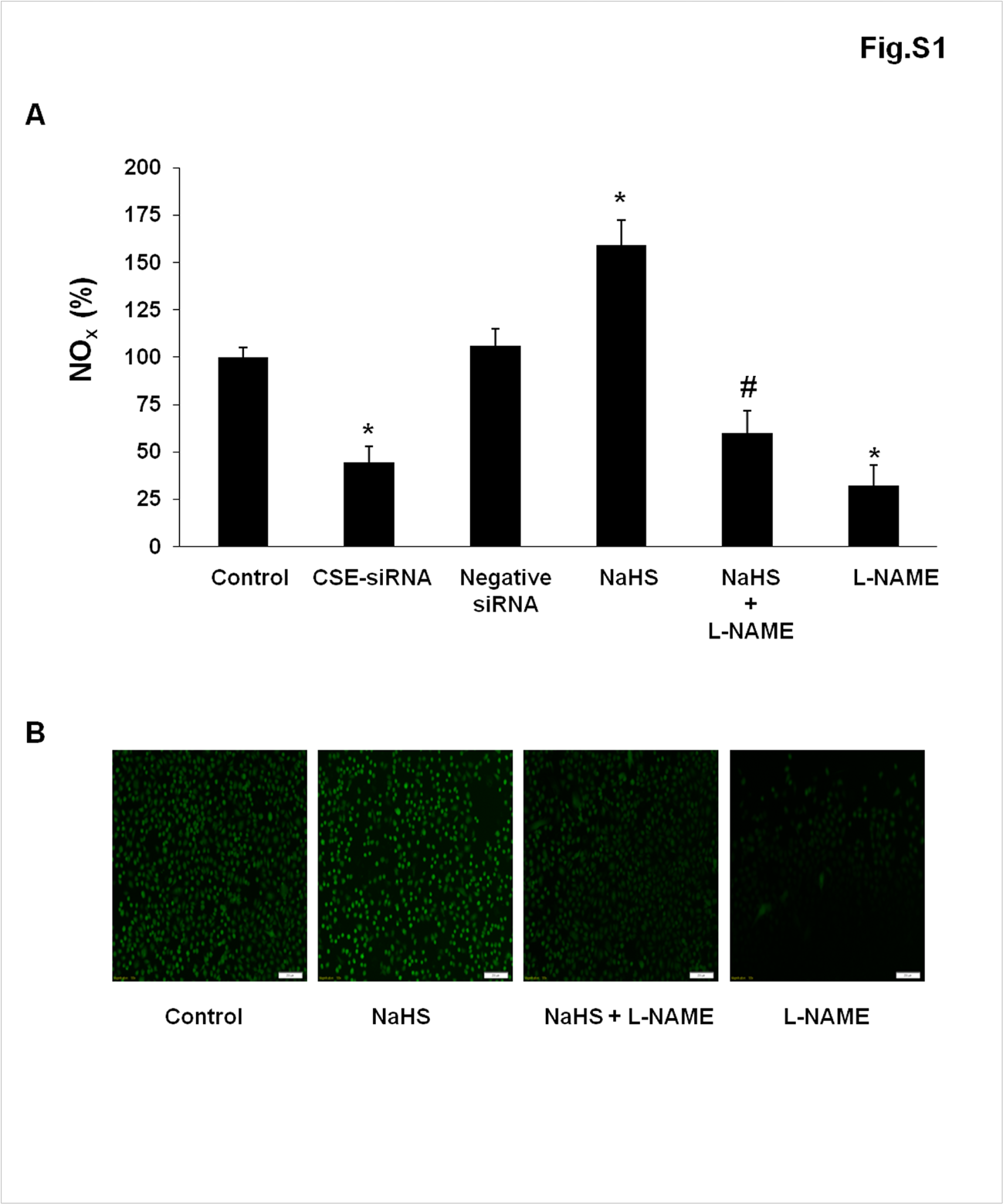

Supplement: Supplementary file 1 [file jcmm0017-0879-SD1.tif]

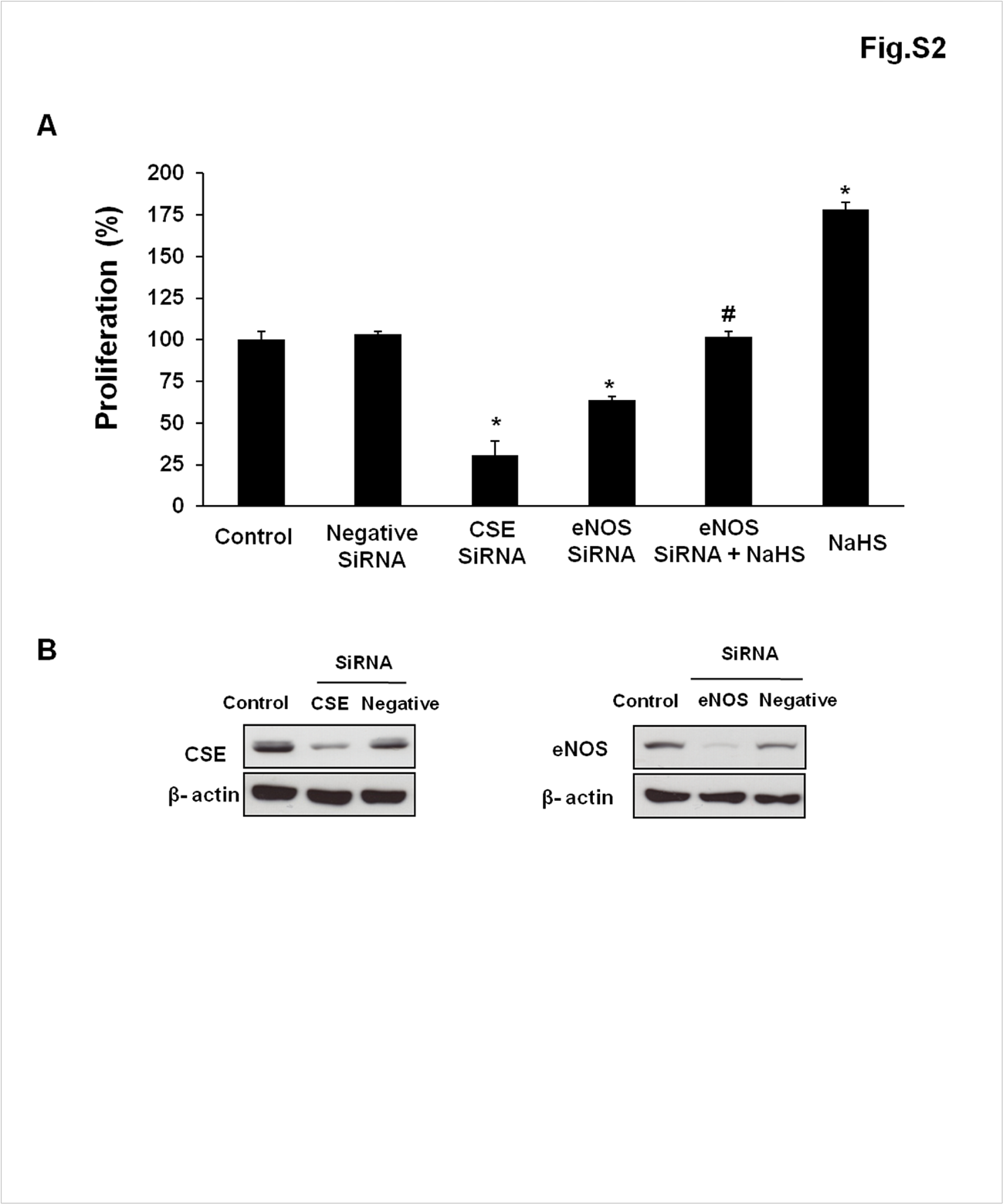

Supplement: Supplementary file 2 [file jcmm0017-0879-SD2.tif]
